# The Value of SII in Predicting the Mortality of Patients with Heart Failure

**DOI:** 10.1155/2022/3455372

**Published:** 2022-05-19

**Authors:** Miao Yuan, Fuxian Ren, Dengfeng Gao

**Affiliations:** ^1^Department of Cardiovascular Disease, Second Affiliated Hospital of Xi'an Jiaotong University, 710000, China; ^2^Department of Cardiology, Meishan Brach of the Third Affiliated Hospital, Yanan University School of Medical, 620000, China

## Abstract

**Background:**

The main purpose of this study was to explore the predictive value of the systemic immune inflammation index (SII), a novel clinical marker, in heart failure (HF) patients.

**Methods:**

Critically ill patients with HF were identified from the Medical Information Mart for Intensive Care III (MIMIC III) database. Patients were divided into three groups according to tertiles of SII (group 1, group 2, group 3). We used Kaplan-Meier curves and Cox proportional hazards regression models to evaluate the association between the SII and all-cause mortality in HF. Subgroup analysis was used to verify the predictive effect of the SII on mortality.

**Results:**

This study included 9107 patients with a diagnosis of HF from the MIMIC III database. After 30, 60, 180, and 365 days of follow-up, 25.60%, 32.10%, 41.30%, and 47.50% of the patients in group 3 had died. Using the Kaplan-Meier curve, we observed that patients with higher SII values had a shorter survival time (log rank *p* < 0.001). The Cox proportional hazards regression model adjusted for all possible confounders and indicated that the higher SII group had a higher mortality (30-day: HR = 1.304, 95%CI = 1.161 − 1.465, 60-day: HR = 1.266, 95% CI = 1.120 − 1.418, 180-day: HR = 1.274, 95%CI = 1.163 − 1.395, and 365-day: HR = 1.255, 95%CI = 1.155 − 1.364).

**Conclusions:**

SII values could be used as a predictor of prognosis in critically ill patients with HF.

## 1. Introduction

Heart failure (HF) is a complex heart disease that is estimated to affect 38 million people worldwide [1]. HF refers to the inability of the heart to fill and eject enough blood to meet the demands of the body. The common clinical symptoms include dyspnea, fatigue, and edema [2]. Although there are many clinical markers that can help predict the prognosis of patients with heart failure [3], further exploration is needed to find more convenient and simple indicators.

The systemic immune-inflammation index (SII) is an indicator of inflammation. It is calculated by *P* × *N*/*L* ratio (SII = *P* × *N*/*L* ratio), where *P* and *N*/*L* are the peripheral platelet count and neutrophil-to-lymphocyte ratio, respectively. At present, studies have shown that there is a certain correlation between the SII and the prognosis of various cancers [4–7].

There is a close relationship between inflammation and cardiovascular disease [8]. Related studies have shown that inflammatory factors can participate in the occurrence and development of atherosclerosis [9] and have a certain relationship with heart failure and cardiac remodeling [10, 11]. The SII, an indicator of inflammation, reflects the level of inflammation and is related to the progression and prognosis of stroke or cardiovascular diseases. Hou et al. found that the SII, an independent risk factor, was associated with stroke severity or prognosis. Jin et al. investigated associations between the SII and the risks for CVDs and all-cause mortality. Their study confirmed that an elevated SII increased the risk of all-cause death [12]. Yang et al. examined the predictive value of the SII in coronary artery disease (CAD) patients and found that it predicted major cardiovascular events better than traditional risk factors in CAD patients after coronary intervention [13]. The predictive value of the SII in HF patients is unclear. In addition, based on previous findings, we speculate that there is a potential relationship between the SII and the prognosis of HF. The purpose of this study was to further explore the importance of the SII in the prognosis of HF by using clinical data in the MIMIC III database.

## 2. Materials and Methods

### 2.1. Database

All the data in the current study were extracted from an online international database—Medical Information Mart for Intensive Care III (MIMIC III). The version of database is 1.4, which contains deidentified patient data from the Beth Israel Deaconess Medical Center for the period from 2001 to 2012 [14]. All the patients in the database were deidentified for privacy protection, and the need for informed consent was waived. The Institutional Review Boards at both Massachusetts Institute of Technology and the Beth Israel Deaconess Medical Center approved the use of the data for research. The database can be accessed by certified researchers; so, no additional informed consent of the patient and ethic approvement is required. The certified researcher of this study is Miao Yuan (no. 7382002).

### 2.2. Inclusion and Exclusion Criteria

Adult patients with a diagnosis of HF according to the International Classification of Diseases (ICD)-9 code were selected (ICD-9 codes 39891, 40201, 40211, 40291, 40401, 40403, 40411, 40413, 40491, 40493, 4280, 4281, 42820-42823, 42830-42833, 42820-42843, 4289). Patients who were younger than 18 years of age were lacking an SII value , had any type of leukemia (ICD-9 codes 20310-20312, 20400-20412, 20410-20412, 20421-20422, 20480-20482, 20490-20492, 20500-20502, 20510-20512, 20520-20522, 20580-20582, 20590-20592, 20600-20602, 20610-20612, 20620-20622, 20680-20682, 20690-20292), and were excluded. For patients who were admitted to the ICU more than once, only the first ICU stay was considered.

### 2.3. Data Extraction

We used Structured Query Language (SQL) and PostgreSQL software (the PostgreSQL Global Development Group, version 9.6) to obtain data from MIMIC III. These data included demographic characteristics (age, sex, ethnicity, marital type), Sequential Organ Failure Assessment (SOFA) score, vital signs (heart rate, systolic pressure, diastolic pressure, respiration rate, temperature, SpO_2_), comorbidities (infection, diabetes, dyslipidemia, hypertension, chronic pulmonary disease, pulmonary circulation, cardiac arrhythmias, valvular disease, peripheral vascular disease, cerebral ischemia, renal failure, liver disease, obesity), the primary disease that causes heart failure (rheumatoid heart disease, coronary heart disease, pulmonary heart disease), the type of heart failure (heart failure with reduced ejection fraction [HFrEF], heart failure with preserved ejection fraction [HFpEF], unknown type), laboratory results (anion gap, creatine, chloride, glucose, hemoglobin, international normalized ratio [INR], prothrombin time [PT], sodium, potassium, blood urea nitrogen [BUN]), and hospital stay (length of hospital stay, length of ICU stay). The SII value was calculated by *P* × *N*/*L* ratio (SII = *P* × *N*/*L* ratio), where *P* and *N*/*L* are the peripheral platelet count and neutrophil-to-lymphocyte ratio, respectively. We extracted vital signs and laboratory results within 24 hours after admission, and if patients had multiple results in the first 24 hours, we took their average.

In our study, 30-day, 60-day, 180-day, and 365-day all-cause mortality were the endpoints. Furthermore, all variables missing more than 20% of the values were deleted. For other continuous variables that were missing fewer than 20% of the values and that were normally distributed, the missing values were replaced by the mean values. Other missing values were replaced by the median values.

### 2.4. Statistical Analysis

We used the Kolmogorov-Smirnov test to test whether continuous variables conformed to a normal distribution. Those that conformed to a normal distribution are expressed as the mean and standard deviation, while those that did not conform to a normal distribution are expressed as the median and quartile spacing. Classification variables were represented by the number or percentage. The patients were divided into three groups (group 1, group 2, group 3) according to tertiles of SII values. We used the chi-square test, one-way ANOVA, and the Kruskal-Wallis H test to identify whether there were significant differences among the groups. The multicollinearity of variables was tested by the variance inflation factor (VIF), and a VIF > 5 was considered indicative of multicollinearity. Kaplan-Meier curves and Cox proportional hazards regression models were used to analyze the relationship between the SII and all-cause mortality at 30 days, 60 days, 180 days, and 365 days, which is expressed by hazard ratio (HR) and 95% confidence intervals (CI). The confounders included clinical comorbidities and laboratory parameters. Then, subgroup analyses were performed to verify the role of the SII in 30-day, 60-day, 180-day, and 365-day all-cause mortality.

Demographic characteristics, comorbidities, and laboratory tests that were significantly different between the three groups were incorporated as confounders. The confounders were included in the Cox proportional hazards regression model. Group1 was used as the reference. We defined three models according to the confounders included. No confounders were included in the unadjusted model. In model I, only age, ethnicity, sex, and marital status were adjusted. In model II, age, ethnicity, sex, marital status, heart rate, diastolic pressure, respiratory rate, SpO_2_, infection, diabetes, dyslipidemia, chronic pulmonary disease, pulmonary circulation, cardiac arrhythmias, liver disease, obesity, anion gap, creatinine, BUN, chloride, hemoglobin, and potassium were adjusted. Subgroup analyses were performed using Cox regression models. The relationship between different levels of the SII and 30-day, 60-day, 180-day, and 365-day all-cause mortality was explored by subgroup analyses of clinical comorbidities primary disease and laboratory parameters. For all tests, *p* < 0.05 was defined as statistically significant in this analysis.

## 3. Results

### 3.1. Subject Characteristics

**Figure 1:**
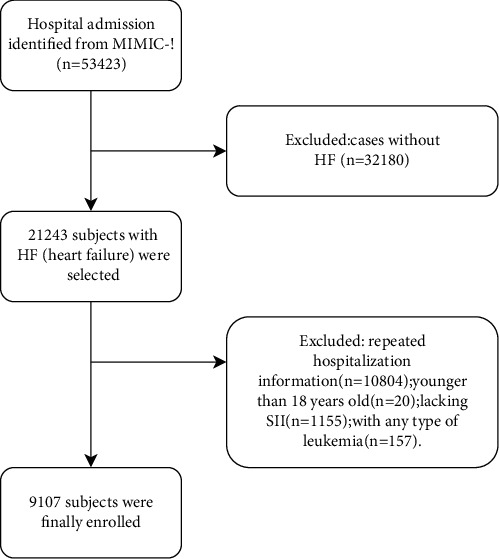
Cohort construction flow chart.

According to the abovementioned selection criteria, 9107 patients were eventually enrolled in this study with a diagnosis of HF (Figure 1). They were divided into three groups (group 1, group 2, group 3) according to tertiles of SII values. The baseline characteristics of the study population are listed in Table 1. Older patients had higher SII values (*p* < 0.001). The proportions of males in group 1 and group 2 were higher, and the proportion of females in group 3 was higher (*p* < 0.001). There was no significant difference in the SOFA score among the three groups (*p* = 0.212), suggesting that there was no significant difference in disease severity among the three groups at admission.

Table 1 Baseline characteristics of the study population by the SII.

Comparing the comorbidities among the three groups, patients with higher SII values tended to have a history of diabetes (*p* = 0.001), chronic pulmonary disease (*p* < 0.001), and cardiac arrhythmia (*p* < 0.001). Patients with higher SII values did not tend to have dyslipidemia (*p* = 0.001), liver disease (*p* < 0.001), or obesity (*p* < 0.001). There was no statistical difference in the proportion of pulmonale heart disease among the three groups (*p* = 0.216). The prevalence of coronary heart disease was lower in the group with higher SII (*p* < 0.001). In addition, we found that there were no statistical differences in the proportion of rheumatic heart disease (*p* = 0.277), but we think the results were not significant because of the small sample size. There were statistical differences in the types of heart failure among the groups (*p* = 0.008). The patients with higher SII values were more likely to have concomitant infection (*p* = 0.001). Moreover, patients with higher SII values (group 3) had higher heart rate (*p* < 0.001) and respiratory rates (*p* < 0.001) and lower diastolic pressure (*p* = 0.019) and SpO2 (*p* < 0.001) than those in group 1 and group 2. Patients with higher SII values had higher anion gap (*p* < 0.001), creatinine (*p* = 0.012), BUN (*p* < 0.001), and hemoglobin (*p* = 0.014) and lower chloride (*p* < 0.001) and potassium (*p* = 0.043). Furthermore, patients with higher SII values seemed to have more severe HF and had longer ICU stays (*p* < 0.001) and hospital stays (*p* < 0.001). Their 30-day mortality, 60-day mortality, 180-day mortality, and 365-day mortality rates were also higher (*p* < 0.001).

### 3.2. Association between All-Cause Mortality and the SII

**Figure 2:**
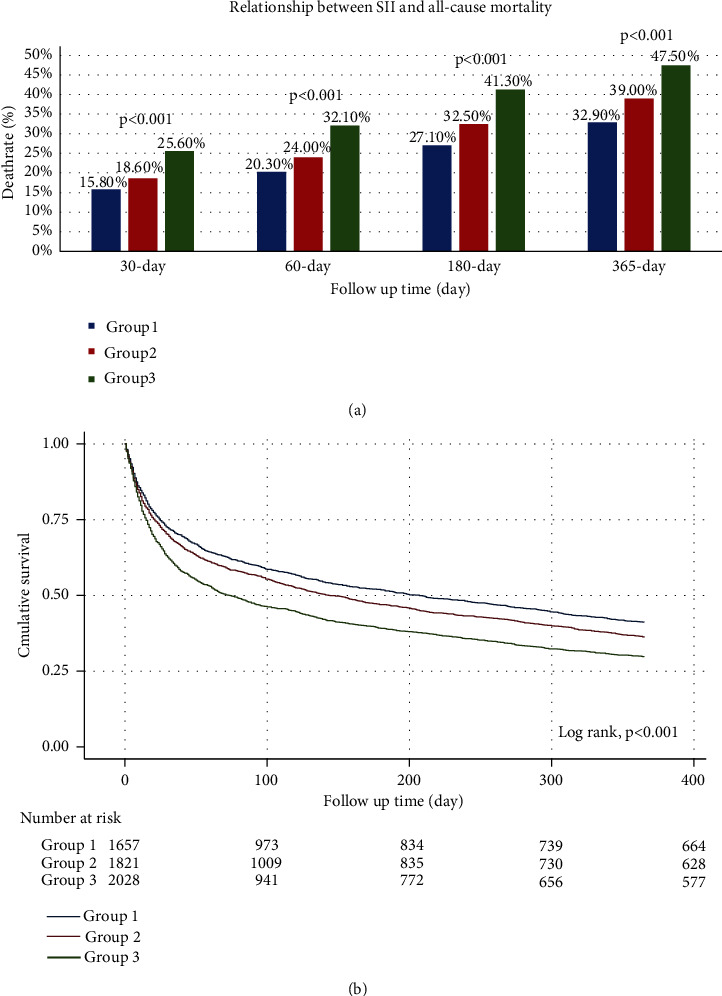
Relationship between the SII and all-cause mortality: (a) the death rate at each timepoint according to tertiles of SII values. (b) Kaplan-Meier survival curve of overall survival in HF patients with different SII values.

Figure 2(a) shows that patients with higher SII values had increased mortality at different timepoints (*p* < 0.001). A positive correlation was observed.  Figure 2(b) shows the Kaplan-Meier curve among the three groups of different SII values. The results indicated that group 3 had the highest all-cause mortality. Patients with higher SII values had an increased risk of death (log rank *p* < 0.001). The multicollinearity test showed that there was no multicollinearity between the confounders (VIF < 5). This result is shown in sTable-1.

In Table 2, a Cox proportional hazards regression model was used to determine the associations between the SII and all-cause mortality at 30 days and 365 days in HF patients. Group 1 was used as reference. In the unadjusted model, Cox proportional hazards regression was used to compare the death risk of the three groups. Model I was adjusted only for age, ethnicity, sex, and marital status. In model II, age, ethnicity, sex, marital status, heart rate, diastolic pressure, respiratory rate, SpO2, infection, diabetes, dyslipidemia, chronic pulmonary disease, pulmonary circulation, cardiac arrhythmias, liver disease, obesity, anion gap, creatinine, BUN, chloride, hemoglobin, and potassium were all adjusted. After adjustment for different confounders, the association between the SII and all-cause mortality remained statistically significant (model I, *p* trend <0.001; model II, *p* trend <0.001). In group 2, the value of the SII in predicting long-term prognosis was better than that in predicting short-term prognosis (unadjusted, 30-day: HR 1.091, 95% CI 0.966-1.232, 365-day: HR 1.131, 95% CI 1.039-1.230; model I, 30-day: HR 1.064, 95% CI 0.942-1.202, 365-day: HR 1.118, 95% CI 1.027-1.217; model II, 30-day: HR 1.037 95% CI 0.916-1.174, 365-day: HR 1.085 95% CI 0.996-1.182). In group 3, the value of the SII in predicting the mortality of patients with HF remained stable with the extension of follow-up time (unadjusted, 30-day: HR 1.395, 95% CI 1.245-1.563, 365-day: HR 1.369, 95% CI 1.263-1.484; model I, 30-day: HR 1.358, 95% CI 1.211-1.523, 365-day: HR 1.353 95% CI 1.247-1.467; model II, 30-day: HR 1.230 95% CI 1.093-1.385, 365-day: HR 1.233 95% CI 1.134-1.341). The similar results were observed of the 60-day and 180-day all-cause mortality (sTable-2).

Table 2 HR (95% CI) for all-cause mortality at 30 days and 365 days across groups.

### 3.3. Subgroup Analysis

**Figure 3:**
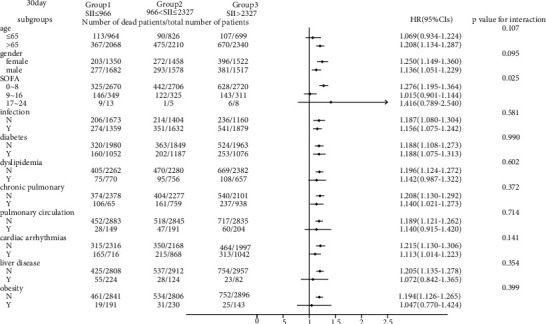
Subgroup analyses of associations between different SII values and 30-day all-cause mortality based on different comorbidities.

**Figure 4:**
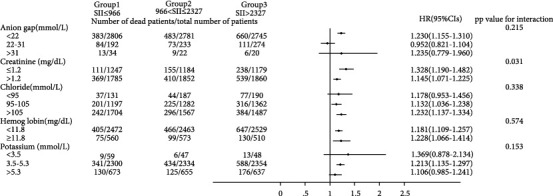
Subgroup analyses of associations between different SII values and 30-day all-cause mortality based on laboratory values.

**Figure 5:**
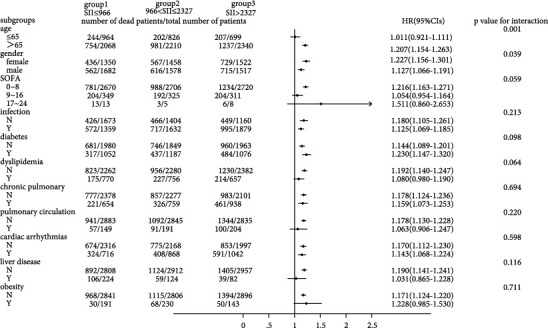
Subgroup analyses of associations between different SII values and 365-day all-cause mortality based on different comorbidities.

**Figure 6:**
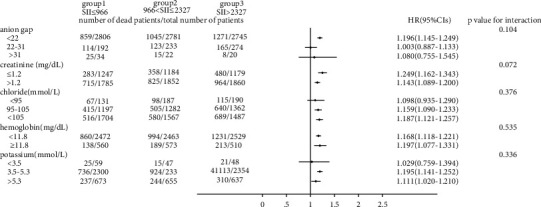
Subgroup analyses of associations between different SII values and 365-day all-cause mortality based on laboratory values.

**Figure 7:**
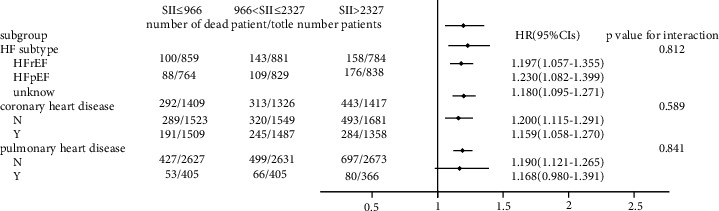
Subgroup analyses of associations between different SII values and 30-day all-cause mortality based on primary disease and the type of HF.

**Figure 8:**
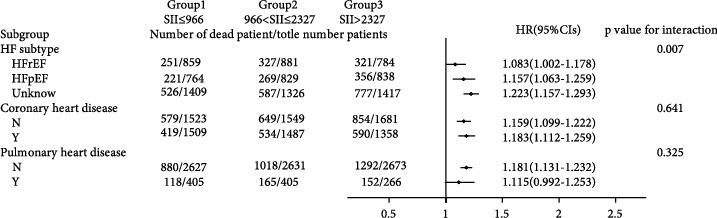
Subgroup analyses of associations between different SII values and 365-day all-cause mortality based on primary disease and the type of HF.

As shown in Figures 3 and 4, we observed that the 30-day all-cause mortality of different levels of the SII was similar in subgroup analysis based on comorbidity and laboratory parameters, and a significant interaction was observed for SOFA score (*p* = 0.025) and creatinine (*p* = 0.031). Figures 5 and 6 show a significant interaction for 365-day all-cause mortality age (*p* = 0.001) and sex (*p* = 0.039). As shown in Figures 7 and 8 , through the subgroup analysis of primary disease and heart failure types, it was found that there was a statistical difference in the prediction of 30-day and 365-day risk of death by the level of SII. We also analyzed subgroup analysis based on comorbidity, laboratory parameters, primary disease, and heart failure types of 60-day (sFig 1-3) and 180-day (sFig 4-6) all-cause mortality of different levels of the SII and observed that the level of SII has predictive value.

## 4. Discussion

Patients with severe HF have a high mortality rate [15], high symptom burden [16] and poor health-related quality of life [17]; so, it is very meaningful to use effective laboratory indicators to predict the risk of death in patients with HF. The well-known BNP has important value in evaluating the prognosis of patients with heart failure [18]. However, BNP also has an obvious limitation, as it is not easily available in clinical practice. In this regard, some simple indicators have more important significance.

Recently, some studies have found that inflammatory factors can be biomarkers of HF [19], and that some inflammatory factors are involved in the occurrence and development of HF [20]. The SII, which is calculated based on platelet counts and the NLR, is considered to be a clinical marker that reflects patients' inflammatory and immune statuses simultaneously. Yang et al. found that SII values can predict prognosis in patients with coronary artery disease [13]. One of the important features of the SII is that it is easy to obtain in the clinic. In our study, we tried to explore the relationship between the SII and prognosis in patients with HF.

Our results showed that patients with higher SII values were more likely to have infection because the SII is a marker of inflammation. This demonstrated that in patients with HF, a severe inflammatory response has a poor prognosis and may lead to a higher risk of death.

Patients with higher SII values were more likely to have chronic diseases such as diabetes, chronic pulmonary disease, and cardiac arrhythmias, suggesting that these patients may have a more obvious inflammatory response on admission to the hospital. Previous studies also confirmed that these diseases were often associated with inflammation [21–24].

Curiously, we found that patients with higher SII values did not seem to be prone to dyslipidemia and obesity. This finding was not consistent with our previous understanding that dyslipidemia and obesity were often associated with inflammation [25, 26]. We considered that the reasons may be the conditions of patients with higher SII values were more severe, their positive fluid balance may result in biased test results, or severe patients were more likely to be complicated with nutritional and metabolic problems.

We observed that patients with liver disease had lower SII values on admission, which may be due to liver disease complicated with hypersplenism, resulting in decreased SII values. However, due to the limited number of patients with liver disease, the results might be biased.

We found that the risk of death increased over time in different SII levels. We also found that at the same follow-up time, the risk of death of patients with heart failure increased with increasing SII values at admission, including mortality at 30 days, 60 days, 180 days, and 365 days after admission to the ICU. This suggested that at admission, the SII was valuable in predicting the risk of death, even if the follow-up time was different.

According to Cox proportional hazards regression analysis, a higher SII value at admission was an independent predictor of mortality, regardless of whether the confounders were adjusted or not. Therefore, we could conclude that the SII was robust in predicting the prognosis of patients with HF. In addition, we found that a higher SII value was of greater significance in predicting poor prognosis. Moreover, the predictive value of the SII for long-term prognosis was greater than that for short-term prognosis. For a short period of time, only significantly increased SII values (group 3) were significant in predicting the risk of death. With the extension of observation time, both mildly increased SII values (group 2) and significantly increased SII values (group 3) were associated with poor prognosis. This suggests that the SII performed better in predicting long-term prognosis.

Through the Kaplan-Meier curve, we observed that with the extension of time, the patients with higher SII values had a higher risk of death. There were significant differences in the risk of death among the three groups.

To verify whether the predictive effect was influenced by cofounders, we conducted interaction and subgroup analyses of the relationship between SII levels and 30-day, 60-day, 180-day, and 365-day all-cause mortality. We found that there were differences in HR values among the subgroup analyses of SOFA score, dyslipidemia, pulmonary circulation, liver disease, obesity, anion gap, and chloride. For patients younger than 65 with higher SOFA scores, dyslipidemia, pulmonary circulation, liver disease, obesity, higher levels of anion gap, and lower levels of chloride, the predictive value of the SII for prognosis was limited. These conditions need to be considered when using the SII to predict the risk of death in HF patients. However, we found that the number of patients in these subgroups was not large; so, we were more inclined to think that these differences resulted from an insufficient number of samples. The difference of primary disease and type of HF did not reflect the predictive value of SII. We did not analyze the subgroup analyses of rheumatoid heart disease, because the number of patients with rheumatoid heart disease was too small.

We found that the interactions between confounders (including age, sex, SOFA score, and anion gap) and the risk of death at different cutoff points were significant. Significant interactions between age and sex with the SII showed that patients with higher SII values were more likely to be elderly and female. The interactions between the SOFA score, anion gap, and the SII tended to be caused by an insufficient sample size.

### 4.1. Limitations

First, our data come from a public database, which is from a single center. More comprehensive clinical data may be needed to further verify the above conclusions in the future. Second, our data were all collected within 24 hours of admission, and not everyone is at the same stage of HF; so, there will be some deviations. Finally, the data in the database had missing values due to bias. As database information is updated and improved, it will be necessary to carry out further research.

## 5. Conclusion

This study shows that SII values can be used as a predictor of prognosis in critically ill patients with HF. Patients with higher values of SII at admission have longer hospital stays and a higher risk of all-cause mortality.

## Figures and Tables

**Table 1:** Baseline characteristics of the study population by SII.

	Group 1	Group 2	Group 3	p value
	SII≤966 n=3032	966<SII≤2327 n=3036	SII>2327 n=3039	
Characteristic				
Age(years)	74(62-83)	75(64-84)	77(66-84)	<0.001
Gender				<0.001
Male	1682(55.5%)	1578(52.0%)	1517(49.9%)	
Female	1350(44.5%)	1458(48.0%)	1522(50.1%)	
SOFA (0-24)	4(2-6)	4(2-6)	4(2-6)	0.212
Ethnicity				<0.001
White	2147(70.8%)	2267(74.7%)	2331(76.7%)	
Asian	58(1.9%)	47(1.5%)	57(1.9%)	
Black	409(13.5%)	243(8.0%)	186(6.1%)	
Hispanic/Latino	92(3.0%)	70(2.3%)	61(2.0%)	
Other	326(10.8%)	409(13.5%)	404(13.3%)	
Marital type				<0.001
Divorced/separated	323(10.7%)	377(12.4%)	346(11.4%)	
Married	1411(46.5%)	1337(44.0%)	1421(46.8%)	
Single	632(20.8%)	574(18.9%)	508(16.7%)	
Widowed	650(21.4%)	720(23.7%)	730(24.0%)	
Other	16(0.5%)	28(0.9%)	34(1.1%)	
Vital sign				
Heartrate(/min)	82.32(72.35-93.38)	83.17(72.65-94.13)	85.73(75.12-96.79)	<0.001
Systolic pressure(mmHg)	113.67(104.72-125.96)	114.97(104.86-126.69)	114.25(104.59-127.56)	0.264
Diastolic pressure(mmHg)	57.12(50.85-63.93)	56.83(50.73-63.68)	56.33(50.34-63.02)	0.019
Respiratory rates(/min)	18.67(16.36-21.31)	19.01(16.83-21.76)	19.69(17.04-22.77)	<0.001
Temperature(°C)	36.74(36.37-37.10)	36.72(36.36-37.11)	36.72(36.33-37.14)	0.467
SpO2(%)	97.37(95.99-98.57)	97.18(95.79-98.44)	97.13(95.64-98.45)	<0.001
Comorbidities				
infection	1359(44.8%)	1632(53.8%)	1879(61.8%)	0.001
diabetes	1052(34.7%)	1187(39.1%)	1076(35.4%)	0.001
dyslipidemia	770(25.4%)	756(24.9%)	657(21.6%)	0.001
hypertension	622(20.5%)	644(21.2%)	620(21.1%)	0.781
chronic pulmonary	654(21.6%)	759(25.0%)	938(30.9%)	<0.001
pulmonary circulation	149(4.9%)	191(6.3%)	204(6.7%)	0.008
cardiac arrhythmias	716(23.6%)	868(28.6%)	1042(34.3%)	<0.001
valvular disease	319(10.5%)	358(11.8%)	359(11.8%)	0.193
peripheral vascular	336(11.1%)	378(12.5%)	379(12.5%)	0.162
cerebral ischemic	131(4.3%)	121(4.0%)	141(4.6%)	0.455
renal failure	747(24.6%)	788(26.0%)	773(25.4%)	0.493
liver disease	224(7.4%)	124(4.1%)	82(2.7%)	<0.001
obesity	191(6.3%)	230(7.6%)	143(4.7%)	<0.001
Primary disease				
rheumatoid heart disease	10(0.33%)	8(0.26%)	4(0.13%)	0.277
pulmonary heart disease	405(13.36%)	405(13.33%)	366(12.04%)	0.216
coronary heart disease	1509(49.77%)	1487(48.99%)	1358(44.69%)	<0.001
The type of heart failure				
HFrEF	859(28.33%)	881(29.02%)	784(25.80%)	
HFpEF	764(25.20%)	829(27.31%)	838(27.57%)	
unknown	1409(46.47%)	1326(43.68%)	1417(46.63%)	
length of hospital stay(day)	8.28(5.13-13.83)	8.85(5.20-14.46)	9.31(5.80-16.64)	<0.001
length of ICU stay(day)	2.68(1.35-5.00)	2.86(1.44-5.50)	3.10(1.72-6.72)	<0.001
Laboratory Results				
anion gap(mmol/L)	15.00(13.00-18.00)	16.00(13.00-18.00)	16.00(14.00-19.00)	<0.001
creatinine(mg/dL)	1.30(0.90-2.00)	1.29(1.00-2.20)	1.40(0.90-2.20)	0.012
BUN(mg/dL)	27.00(18.00-43.00)	29.00(20.00-48.00)	31.00(21.00-50.00)	<0.001
chloride(mmol/L)	106.00(102.00-110.00)	106(101-110)	105(101-109)	<0.001
glucose(mg/dL)	163(128-205)	164.00(128.00-210.00)	165.00(129.00-214.00)	0.152
hemoglobin(mg/dL)	9.70(8.40-11.20)	9.90(8.60-11.30)	9.90(8.60-11.10)	0.014
INR	1.50(1.20-1.90)	1.50(1.20-1.90)	1.50(1.20-1.90)	0.293
PT(seconds)	15.70(13.80-18.49)	15.50(13.70-18.49)	15.60(13.70--18.49)	0.203
sodium(mmol/L)	140.00(138.00-142.00)	140.00(137.00-142.00)	140.00(138.00-143.00)	0.774
potassium(mmol/L)	4.60(4.20-5.20)	4.60(4.20-5.20)	4.60(4.10-5.20)	0.043
30-day mortality	480(15.8%)	565(18.6%)	777(25.6%)	<0.001
60-day mortality	615(20.3%)	730(24.0%)	974(32.1%)	<0.001
180-day mortality	821(27.1%)	986(32.5%)	1254(41.3%)	<0.001
365-day mortality	998(32.9%)	1183(39.0%)	1444(47.5%)	<0.001

INR: international normalized ratio; PT: prothrombin time; BUN: blood urea nitrogen; HFrEF: heart failure with reduced ejection fraction; HFpEF: heart failure with preserved ejection fraction.

**Table 2:** HR (95% CI) for all-cause mortality of 30-day and 365-day across groups.

Variable	Non-adjusted	P value	Model I	P value	Model II	P value
	HR (95% CIs)		HR (95% CIs)		HR (95% CIs)	
30-day all-cause mortality						
SII group (tertiles)						
Group1	1		1		1	
Group2	1.091(0.966-1.232)	0.161	1.064(0.942-1.202)	0.320	1.037(0.916-1.174)	0.569
Group3	1.395(1.245-1.563)	<0.001	1.358(1.211-1.523)	<0.001	1.230(1.093-1.385)	0.001
**P trend**		<0.001		<0.001		<0.001

365-day all-cause mortality						
SII group (tertiles)						
Group1	1		1		1	
Group2	1.131(1.039-1.230)	0.004	1.118(1.027-1.217)	0.010	1.085(0.996-1.182)	0.043
Group3	1.369(1.263-1.484)	<0.001	1.353(1.247-1.467)	<0.001	1.233(1.134-1.341)	<0.001
P trend		<0.001		<0.001		<0.001

## Data Availability

The datasets and data used and/or analyzed during the current study are available from the corresponding authors on reasonable request.

## Supplementary Materials

Supplementary Materials sTable 1: multicollinearity statistics between cofounders. sTable 2: HR (95% CI) for all-cause mortality at 60 days and 180 days across groups. sFig 1: subgroup analyses of associations between different SII values and 60-day all-cause mortality based on different comorbidities. sFig 2: subgroup analyses of associations between different SII values and 60-day all-cause mortality based on laboratory values. sFig 3: subgroup analyses of associations between different SII values and 60-day all-cause mortality based on primary disease and the type of HF. sFig 4: subgroup analyses of associations between different SII values and 180-day all-cause mortality based on different comorbidities. sFig 5: subgroup analyses of associations between different SII values and 180-day all-cause mortality based on laboratory values. sFig 6: subgroup analyses of associations between different SII values and 180-day all-cause mortality based on primary disease and the type of HF.
