# Supplementary material for: The Value of SII in Predicting the Mortality of Patients with Heart Failure
Source: Dis Markers. 2022 May 19;2022:3455372. doi: 10.1155/2022/3455372 (PMC9135558; doi:10.1155/2022/3455372)
Supplement: Supplementary Materials — sTable 1: multicollinearity statistics between cofounders. sTable 2: HR (95% CI) for all-cause mortality at 60 days and 180 days across groups. sFig 1: subgroup analyses of associations between different SII values and 60-day all-cause mortality based on different comorbidities. sFig 2: subgroup analyses of associations between different SII values and 60-day all-cause mortality based on laboratory values. sFig 3: subgroup analyses of associations between different SII values and 60-day all-cause mortality based on primary disease and the type of HF. sFig 4: subgroup analyses of associations between different SII values and 180-day all-cause mortality based on different comorbidities. sFig 5: subgroup analyses of associations between different SII values and 180-day all-cause mortality based on laboratory values. sFig 6: subgroup analyses of associations between different SII values and 180-day all-cause mortality based on primary disease and the type of HF. [file 3455372.f1.zip › 3455372.f1/sTable 1.pdf]

sTable-1
Multicollinearity statistics between cofounders

Model	Beta	t	Sig.	Collinearity	
				Tolerance	VIF
**gender**	-66.987	-.668	.504	.853	1.173
**marital type**	45.998	.931	.352	.887	1.127
**ethnicity**	-55.049	-1.655	.098	.964	1.037
**age**	-.673	-.165	.869	.680	1.471
**heartrate**	21.002	5.934	.000	.690	1.449
**systolic pressure**	10.018	3.003	.003	.636	1.573
**diastolic pressure**	-26.689	-4.478	.000	.546	1.831
**respiratory rates**	43.873	3.530	.000	.817	1.223
**temperature**	-167.615	-2.151	.032	.855	1.170
**SpO2**	40.872	2.222	.026	.877	1.140
**infection**	649.958	6.774	.000	.934	1.071
**diabetes**	-41.895	-.413	.679	.899	1.112
**dyslipidemia**	-306.145	-2.741	.006	.941	1.063
**hypertension**	62.761	.316	.752	.327	3.061
**chronic pulmonary**	460.872	4.249	.000	.950	1.053
**pulmonary circulation**	18.261	.091	.927	.951	1.051
**cardiac arrhythmias**	321.682	2.892	.004	.843	1.187
**valvular disease**	-178.416	-1.167	.243	.908	1.101
**peripheral vascular**	-75.839	-.523	.601	.964	1.038
**cerebral ischemic**	-222.196	-.963	.336	.973	1.028
**renal failure**	-112.865	-.592	.554	.311	3.213
**liver disease**	-1048.907	-4.709	.000	.958	1.044
**obesity**	-193.956	-.972	.331	.925	1.081
**anion gap**	52.418	4.026	.000	.633	1.580
**creatinine**	-138.765	-3.570	.000	.467	2.140

**BUN**	8.327	3.401	.001	.536	1.865
**hemoglobin**	-39.983	-1.587	.113	.784	1.276
**INR**	4.193	.106	.916	.411	2.436
**PT**	2.676	.414	.679	.407	2.457
**sodium**	.938	.070	.944	.525	1.905
**potassium**	-14.180	-.268	.789	.850	1.176
**chloride**	-26.902	-2.618	.009	.480	2.084
**glucose**	.312	.613	.540	.840	1.191

INR: international normalized ratio; PT: prothrombin time; BUN: blood urea nitrogen
