# Supplementary material for: The Value of SII in Predicting the Mortality of Patients with Heart Failure
Source: Dis Markers. 2022 May 19;2022:3455372. doi: 10.1155/2022/3455372 (PMC9135558; doi:10.1155/2022/3455372)
Supplement: Supplementary Materials — sTable 1: multicollinearity statistics between cofounders. sTable 2: HR (95% CI) for all-cause mortality at 60 days and 180 days across groups. sFig 1: subgroup analyses of associations between different SII values and 60-day all-cause mortality based on different comorbidities. sFig 2: subgroup analyses of associations between different SII values and 60-day all-cause mortality based on laboratory values. sFig 3: subgroup analyses of associations between different SII values and 60-day all-cause mortality based on primary disease and the type of HF. sFig 4: subgroup analyses of associations between different SII values and 180-day all-cause mortality based on different comorbidities. sFig 5: subgroup analyses of associations between different SII values and 180-day all-cause mortality based on laboratory values. sFig 6: subgroup analyses of associations between different SII values and 180-day all-cause mortality based on primary disease and the type of HF. [file 3455372.f1.zip › 3455372.f1/sTable 2.pdf]

sTable-2

HR (95% CI) for all-cause mortality at 60 days and 180 days across groups

Variable	Non-adjusted		Model I		Model II	
**60-day all-cause mortality**	**HR (95% CIs)**	**P value**	**HR (95% CIs)**	**P value**	**HR (95% CIs)**	**P value**
**SII group (tertiles)**	Non-adjusted		Model I		Model II	P value
**group1**	1		1		1	
**group2**	1.107(0.995-1.233)	0.062	1..083(0.972-1.206)	0.148	1.057(0.947-1.180)	0.320
**group3**	1.401(1.266-1.550)	< 0.001	1.369(1.236-1.515)	0.001	1.242(1.118-1.379)	< 0.001
**P trend**		< 0.001		< 0.001		< 0.001
**180-day all-cause mortality**						
**SII group (tertiles)**						
**group1**	1		1		1	
**group2**	1.133(1.032-1.243)	0.008	1.113(1.014-1.222)	0.024	1.081(0.984-1.189)	0.105
**group3**	1.404(1.286-1.534)	< 0.001	1.380(1.263-1.508)	< 0.001	1.255(1.145-1.375)	< 0.001
**P trend**		< 0.001		< 0.001		< 0.001
