# Supplementary figures and images for: The Value of SII in Predicting the Mortality of Patients with Heart Failure
Source: Dis Markers. 2022 May 19;2022:3455372. doi: 10.1155/2022/3455372 (PMC9135558; doi:10.1155/2022/3455372)

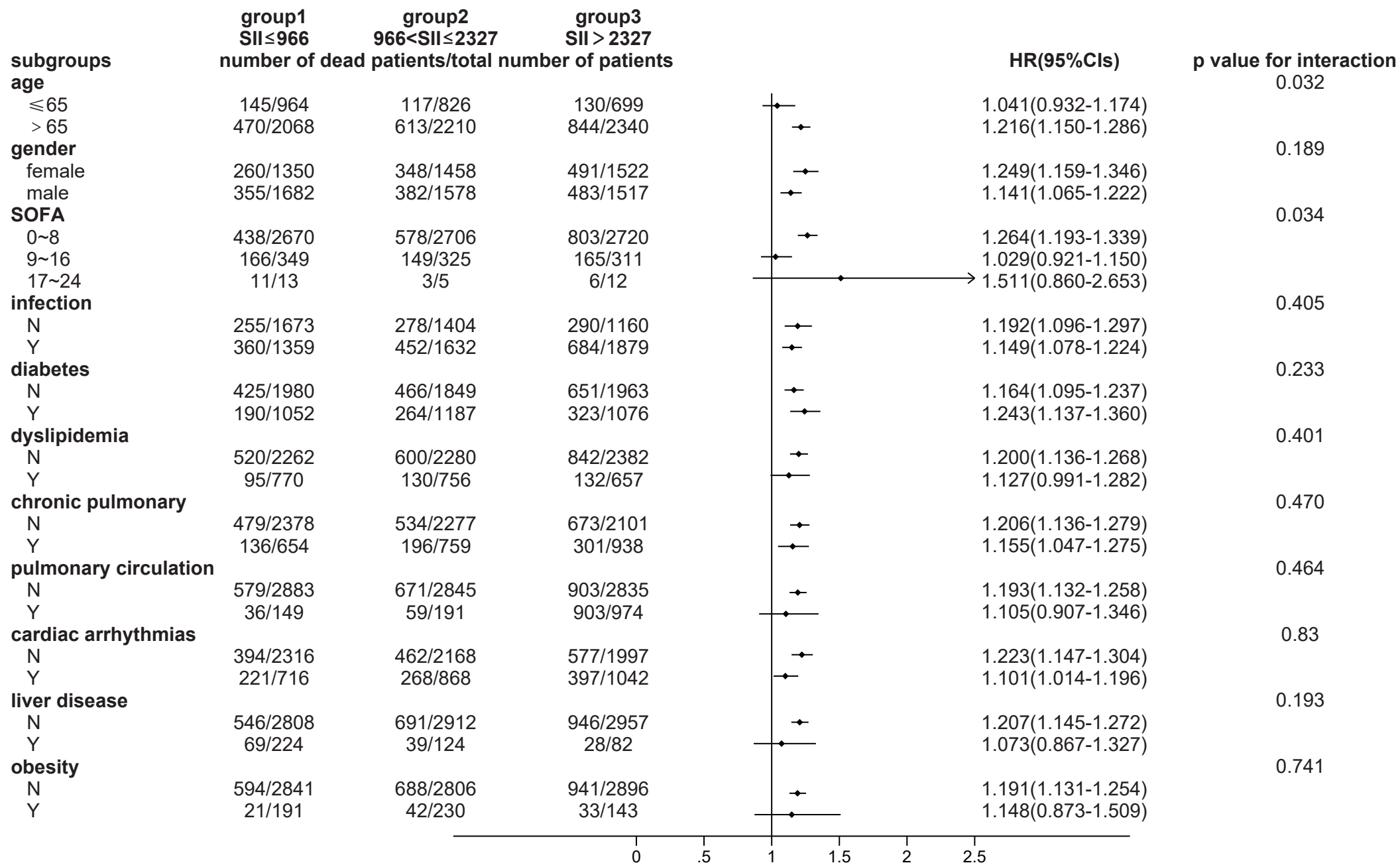

Supplement: Supplementary Materials — sTable 1: multicollinearity statistics between cofounders. sTable 2: HR (95% CI) for all-cause mortality at 60 days and 180 days across groups. sFig 1: subgroup analyses of associations between different SII values and 60-day all-cause mortality based on different comorbidities. sFig 2: subgroup analyses of associations between different SII values and 60-day all-cause mortality based on laboratory values. sFig 3: subgroup analyses of associations between different SII values and 60-day all-cause mortality based on primary disease and the type of HF. sFig 4: subgroup analyses of associations between different SII values and 180-day all-cause mortality based on different comorbidities. sFig 5: subgroup analyses of associations between different SII values and 180-day all-cause mortality based on laboratory values. sFig 6: subgroup analyses of associations between different SII values and 180-day all-cause mortality based on primary disease and the type of HF. [file 3455372.f1.zip › 3455372.f1/sFig1.pdf]

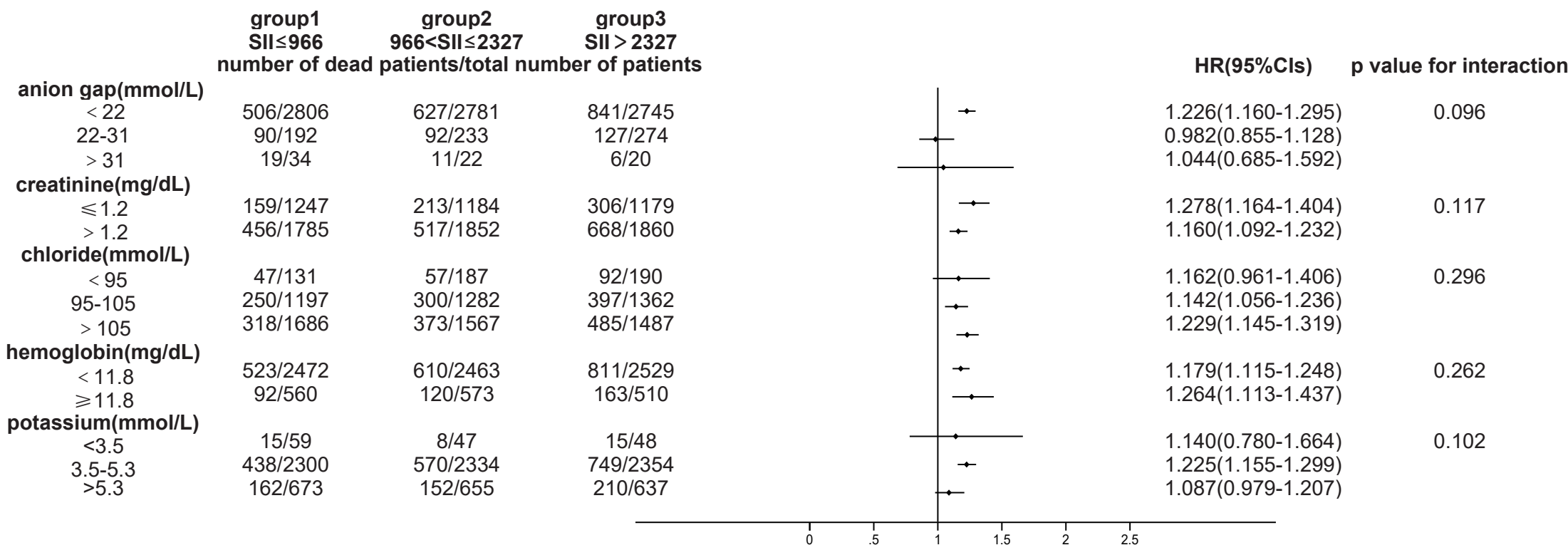

Supplement: Supplementary Materials — sTable 1: multicollinearity statistics between cofounders. sTable 2: HR (95% CI) for all-cause mortality at 60 days and 180 days across groups. sFig 1: subgroup analyses of associations between different SII values and 60-day all-cause mortality based on different comorbidities. sFig 2: subgroup analyses of associations between different SII values and 60-day all-cause mortality based on laboratory values. sFig 3: subgroup analyses of associations between different SII values and 60-day all-cause mortality based on primary disease and the type of HF. sFig 4: subgroup analyses of associations between different SII values and 180-day all-cause mortality based on different comorbidities. sFig 5: subgroup analyses of associations between different SII values and 180-day all-cause mortality based on laboratory values. sFig 6: subgroup analyses of associations between different SII values and 180-day all-cause mortality based on primary disease and the type of HF. [file 3455372.f1.zip › 3455372.f1/sFig2.pdf]

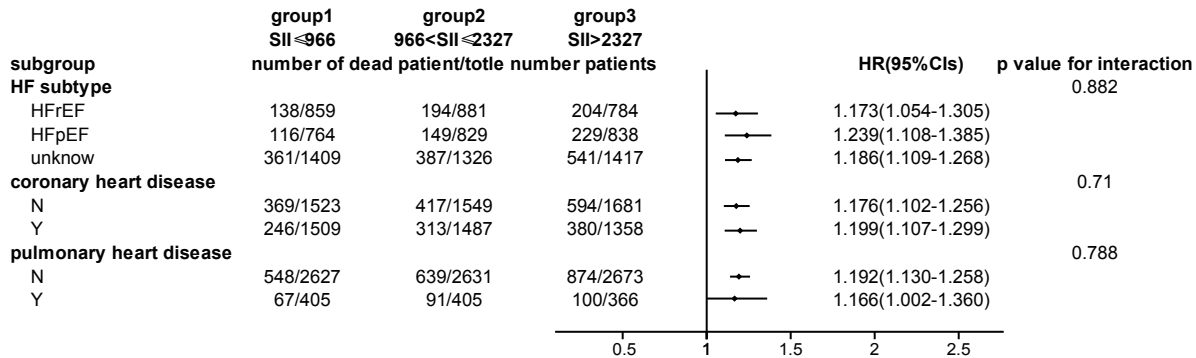

Supplement: Supplementary Materials — sTable 1: multicollinearity statistics between cofounders. sTable 2: HR (95% CI) for all-cause mortality at 60 days and 180 days across groups. sFig 1: subgroup analyses of associations between different SII values and 60-day all-cause mortality based on different comorbidities. sFig 2: subgroup analyses of associations between different SII values and 60-day all-cause mortality based on laboratory values. sFig 3: subgroup analyses of associations between different SII values and 60-day all-cause mortality based on primary disease and the type of HF. sFig 4: subgroup analyses of associations between different SII values and 180-day all-cause mortality based on different comorbidities. sFig 5: subgroup analyses of associations between different SII values and 180-day all-cause mortality based on laboratory values. sFig 6: subgroup analyses of associations between different SII values and 180-day all-cause mortality based on primary disease and the type of HF. [file 3455372.f1.zip › 3455372.f1/sFig3.pdf]

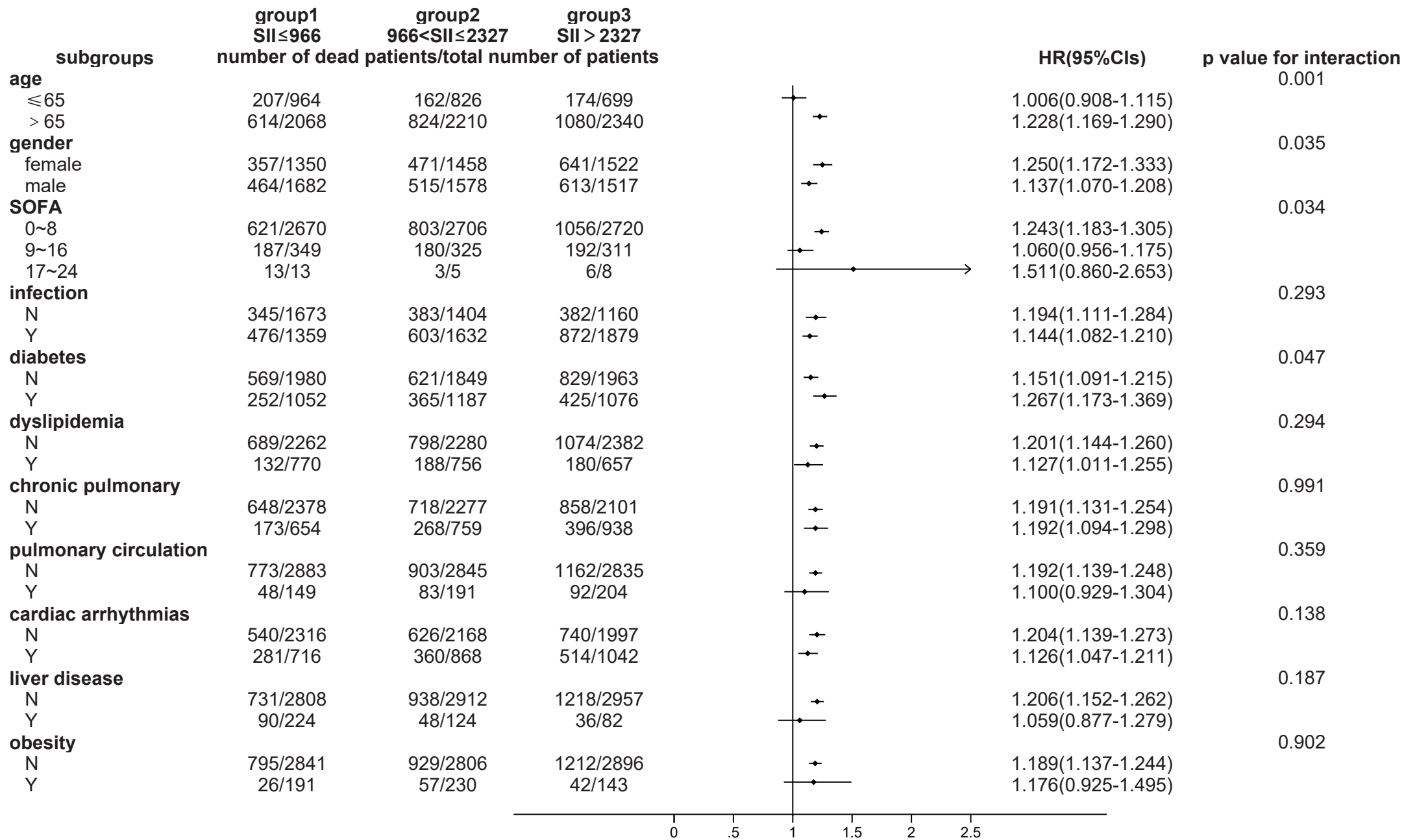

Supplement: Supplementary Materials — sTable 1: multicollinearity statistics between cofounders. sTable 2: HR (95% CI) for all-cause mortality at 60 days and 180 days across groups. sFig 1: subgroup analyses of associations between different SII values and 60-day all-cause mortality based on different comorbidities. sFig 2: subgroup analyses of associations between different SII values and 60-day all-cause mortality based on laboratory values. sFig 3: subgroup analyses of associations between different SII values and 60-day all-cause mortality based on primary disease and the type of HF. sFig 4: subgroup analyses of associations between different SII values and 180-day all-cause mortality based on different comorbidities. sFig 5: subgroup analyses of associations between different SII values and 180-day all-cause mortality based on laboratory values. sFig 6: subgroup analyses of associations between different SII values and 180-day all-cause mortality based on primary disease and the type of HF. [file 3455372.f1.zip › 3455372.f1/sFig4.pdf]

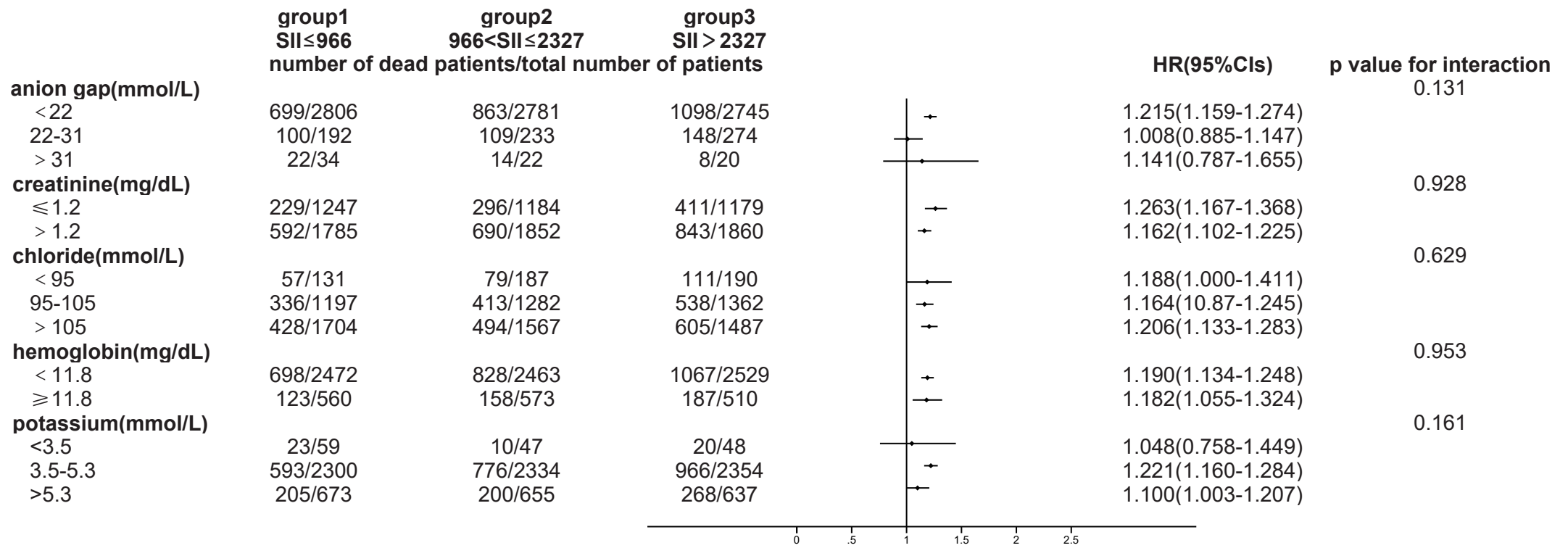

Supplement: Supplementary Materials — sTable 1: multicollinearity statistics between cofounders. sTable 2: HR (95% CI) for all-cause mortality at 60 days and 180 days across groups. sFig 1: subgroup analyses of associations between different SII values and 60-day all-cause mortality based on different comorbidities. sFig 2: subgroup analyses of associations between different SII values and 60-day all-cause mortality based on laboratory values. sFig 3: subgroup analyses of associations between different SII values and 60-day all-cause mortality based on primary disease and the type of HF. sFig 4: subgroup analyses of associations between different SII values and 180-day all-cause mortality based on different comorbidities. sFig 5: subgroup analyses of associations between different SII values and 180-day all-cause mortality based on laboratory values. sFig 6: subgroup analyses of associations between different SII values and 180-day all-cause mortality based on primary disease and the type of HF. [file 3455372.f1.zip › 3455372.f1/sFig5.pdf]

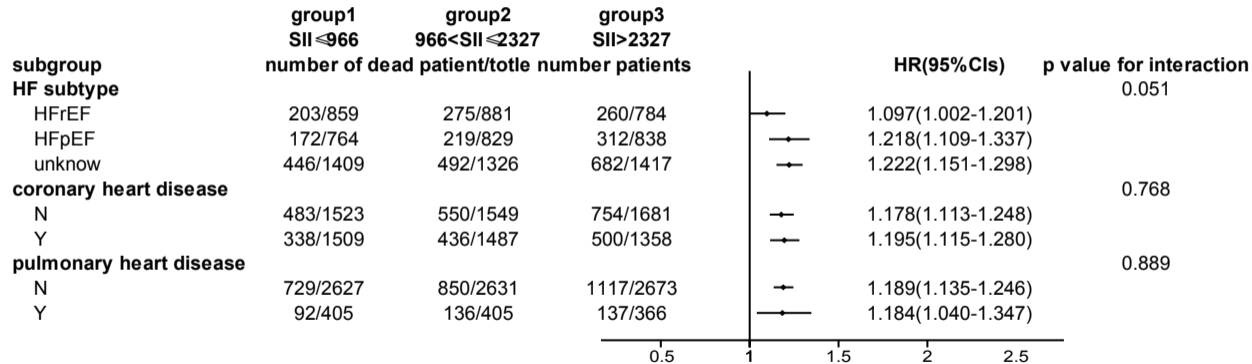

Supplement: Supplementary Materials — sTable 1: multicollinearity statistics between cofounders. sTable 2: HR (95% CI) for all-cause mortality at 60 days and 180 days across groups. sFig 1: subgroup analyses of associations between different SII values and 60-day all-cause mortality based on different comorbidities. sFig 2: subgroup analyses of associations between different SII values and 60-day all-cause mortality based on laboratory values. sFig 3: subgroup analyses of associations between different SII values and 60-day all-cause mortality based on primary disease and the type of HF. sFig 4: subgroup analyses of associations between different SII values and 180-day all-cause mortality based on different comorbidities. sFig 5: subgroup analyses of associations between different SII values and 180-day all-cause mortality based on laboratory values. sFig 6: subgroup analyses of associations between different SII values and 180-day all-cause mortality based on primary disease and the type of HF. [file 3455372.f1.zip › 3455372.f1/sFig6.pdf]
